# Nanoengineering InP Quantum Dot-Based Photoactive Biointerfaces for Optical Control of Neurons

**DOI:** 10.3389/fnins.2021.652608

**Published:** 2021-06-23

**Authors:** Onuralp Karatum, Mohammad Mohammadi Aria, Guncem Ozgun Eren, Erdost Yildiz, Rustamzhon Melikov, Shashi Bhushan Srivastava, Saliha Surme, Itir Bakis Dogru, Houman Bahmani Jalali, Burak Ulgut, Afsun Sahin, Ibrahim Halil Kavakli, Sedat Nizamoglu

**Affiliations:** ^1^Department of Electrical and Electronics Engineering, Koc University, Istanbul, Turkey; ^2^Department of Biomedical Science and Engineering, Koc University, Istanbul, Turkey; ^3^Research Center for Translational Medicine, Koc University, Istanbul, Turkey; ^4^Department of Molecular Biology and Genetics, Koc University, Istanbul, Turkey; ^5^Department of Chemistry, Bilkent University, Ankara, Turkey; ^6^Department of Ophthalmology, Medical School, Koc University, Istanbul, Turkey

**Keywords:** biointerface, neuromodulation, photostimulation, quantum dot, indium phosphide, nanocrystal, neural interface, nanoengineering

## Abstract

Light-activated biointerfaces provide a non-genetic route for effective control of neural activity. InP quantum dots (QDs) have a high potential for such biomedical applications due to their uniquely tunable electronic properties, photostability, toxic-heavy-metal-free content, heterostructuring, and solution-processing ability. However, the effect of QD nanostructure and biointerface architecture on the photoelectrical cellular interfacing remained unexplored. Here, we unravel the control of the photoelectrical response of InP QD-based biointerfaces *via* nanoengineering from QD to device-level. At QD level, thin ZnS shell growth (∼0.65 nm) enhances the current level of biointerfaces over an order of magnitude with respect to only InP core QDs. At device-level, band alignment engineering allows for the bidirectional photoelectrochemical current generation, which enables light-induced temporally precise and rapidly reversible action potential generation and hyperpolarization on primary hippocampal neurons. Our findings show that nanoengineering QD-based biointerfaces hold great promise for next-generation neurostimulation devices.

## Introduction

Neural stimulation offers an effective therapeutic method for the treatment of various health problems. Extracellular stimulation of neurons has led to the development of many prosthetic devices such as artificial retina implants for the treatment of retinal degeneration (Mathieson et al., 2012), cochlear implants for the patients with hearing loss (Moore and Shannon, 2009), and brain stimulation electrodes for treating neurological disorders like anxiety (Sturm et al., 2007), depression (Schlaepfer et al., 2008), and Parkinson’s disease (Benabid et al., 2009). The conventional way for stimulation of neural tissues is through electrical stimulation. Electrodes made of different materials, such as platinum, iridium oxide, titanium nitride, and poly(ethylenedioxythiophene) (PEDOT), have been used for electrical stimulation of neurons and also for recording the electrophysiological activity (Cogan, 2008). Improving the feasibility of such electrodes, while discovering alternative ones for more effective stimulation and recording, has been a topic under extensive research. However, electrical stimulation has several drawbacks including mechanical instability, invasiveness of electrodes, and surgical difficulties due to electrical components.

Instead, optical stimulation provides effective routes for controlling and manipulating the neural activity with high spatiotemporal resolution and less invasive ways. Optogenetics offers a beneficial approach for the photostimulation of neurons; however, its dependence on genetic modification currently limits its use in clinics. Alternatively, photoactive surfaces present a non-genetic way for photostimulation. Biointerfaces utilizing the photoactive surfaces have attracted significant attention in the last two decades due to their high temporal and spatial resolution, ease of fabrication, and effective performance both *in vitro* and *in vivo* (Maya-Vetencourt et al., 2017). Different materials such as organic semiconducting polymers (Ghezzi et al., 2013; Gautam et al., 2014; Abdullaeva et al., 2018; Melikov et al., 2020a), silicon (Jiang et al., 2018), and graphene (Savchenko et al., 2018) have been used as photoactive layers in the biointerfaces. On the other hand, semiconducting quantum dots (QDs) are among the less studied members for photostimulation.

Quantum dots have outstanding properties including band gap tunability due to quantum confinement effect, high photostability, solution processability, and absorption in the visible spectrum, which make them an ideal candidate to be used as a photoactive layer in biointerfaces. Pappas et al. (2007) demonstrated the first QD-based biointerface that utilizes thin films of HgTe QDs as a photoactive layer for the photostimulation of neurons. Later, Lugo et al. (2012) reported cellular interfaces with thin films of CdTe and CdSe QDs as photoactive layers that can make neurons fire action potentials. Those studies by Pappas et al. (2007) and Lugo et al. (2012) make use of QDs that include cadmium and mercury, which raises concerns about the biocompatibility of biointerfaces. Previously, our group showed successful operation of neural stimulation devices based on a biocompatible photoactive layer of InP/ZnO core/shell QDs (Bahmani Jalali et al., 2018b). More recently, we demonstrated a quantum funnel structure based on InP-based QDs, which can enhance the photocurrent production of QD-based biointerfaces through the non-radiative energy transfer mechanism (Bahmani Jalali et al., 2019a). Different from those studies, this report presents device- and nanostructure-level engineering to control the direction and strength of the neural modulation, leading to temporally precise, and rapidly reversible photostimulation of neurons.

Studies showed that both inhibition and stimulation of neural activity can provide a useful toolbox against neurological diseases. On one side, hyperpolarization of neural membrane can lead to inhibition of the activity of neurons and thus suppression of neurological disorders such as epileptic seizures. On the other side, increasing neural activity through low frequency or high frequency stimulation of neurons as in the case of deep brain stimulation is an effective and clinically approved tool for the treatment of certain neurological disorders such as Parkinson’s disease and depression (Benabid, 2003; Mayberg et al., 2005; Sada et al., 2015). Hence, biointerfaces that can perform hyperpolarization and depolarization in a controlled fashion can be effective for therapeutic purposes. To that end, we fabricated biointerfaces based on a biocompatible photoactive layer of InP/ZnS core/shell QDs and an intermediate layer of metal oxide nanoparticles. We designed two different device architectures, namely, type I and type II, to achieve bidirectional stimulation and compared their performances by analyzing their photoelectrical responses to determine the most effective configurations. After having bidirectional photoresponse, we optimized the nanostructure of the photoactive layer by comparing the performances of only InP core QDs and InP/ZnS core/shell QDs. Moreover, we explored the correlation between photocurrent and photoactive layer thickness in the biological medium by conducting electrochemical experiments in artificial cerebrospinal fluid (aCSF), which revealed the optimum photoactive layer thickness maximizing the photoelectrical response. The electrophysiology recordings confirmed the successful photoelectrical coupling of the biointerfaces to neural membrane that allows optical control of the electrical activity of primary hippocampal neurons. Thanks to the nanoengineering of the photoactive biointerfaces, while type II biointerfaces induce depolarization of neural membrane and evoke recurring action potentials, type I biointerfaces hyperpolarize the neural membrane.

## Materials and Methods

### InP Core and InP/ZnS Core/Shell QD Synthesis

InP/ZnS QDs with one monolayer shell were synthesized by hot injection method (Bahmani Jalali et al., 2018a). For the core synthesis, firstly, 56 mg (0.01 mmol) stearic acid (SA), 86 mg zinc undecylenate (0.01 mmol), and 96 mg (0.2 mmol) hexadecylamine (HDA) were mixed in a three-neck flask with 6 ml 1-octadecene (ODE). Afterward, 44 mg (0.1 mmol) indium chloride (InCl_3_) was added into the solution in nitrogen atmosphere. The solution was heated to 120°C and evacuated 20 min in order to provide oxygen and water-free reaction medium. Then, the solution was refilled with a nitrogen atmosphere and heated to 230°C. At this temperature, 1 ml of Tris(trimethylsilyl) phosphine P(TMS)_3_ stock solution (0.2 mmol) was injected to the solution and it was kept at 230°C 20 min. Before the shelling process, the solution was cooled down to room temperature and half of the solution was taken and labeled as core solution. For preparing InP/1ZnS at room temperature, 54 mg zinc diethyldithiocarbamate (0.15 mmol) and 2 ml ODE were added into the solution, respectively. After that, the solution was heated up to 180°C and stirred 30 min. The solution was cooled down to room temperature and purified by washing toluene and ethanol. At the final stage, QDs were re-dispersed in toluene.

### ZnO Nanoparticle Synthesis

ZnO nanoparticles were synthesized using a previously reported method (Karatum et al., 2019). Tetramethylammonium hydroxide (TMAH) dissolved in ethanol (0.55 M) was slowly added to the solution of zinc acetate dihydrate dissolved in dimethyl sulfoxide (DMSO) (0.5 M). After 1 h stirring at room temperature, the solution was washed twice, and dispersed in ethanol at a concentration of 50 mg ml^–1^.

### Biointerface Fabrication

The ITO coated glass substrates were first cleaned by sonicating in detergent solution, deionized water, acetone, and isopropanol consecutively for 15 min each. The cleaned substrates were applied 15 min of UV ozone treatment before moving to layer depositions. TiO_2_ layer on ITO was formed using a commercially available TiO_2_ paste (Sigma-Aldrich) by doctor blading followed by annealing at 400°C for 1 h. ZnO layer was deposited by spin-coating the 50 mg ml^–1^ ZnO nanoparticle solution at 2000 rpm and baked at 100°C for 30 min. The InP/ZnS core/shell QD film was formed by spin coating its 60 mg ml^–1^ solution in toluene at 2000 rpm. For multilayer coating, each layer was treated with 3-mercaptopropionic acid in methanol and then rinsed with methanol, both spin-cast at 2000 rpm, before moving to the coating of next QD layer. After the multilayer coating, the QD film was baked at 100°C for 30 min. The layer thicknesses were characterized by atomic force microscopy (AFM, Bruker, Dimension Icon) in tapping mode with three different scan sizes (40 × 40 μm^2^, 20 × 20 μm^2^, 10 × 10 μm^2^).

### Optical Characterization

UV/visible absorption and photoluminescence (PL) spectra of InP core and InP/ZnS core/shell QDs were obtained using Edinburgh Instruments Spectrofluorometer FS5. Quantum yield measurements were conducted in the integrating sphere module of FS5.

### Photoresponse Analysis

The photocurrent/photovoltage response of our biointerfaces was measured with Autolab Potentiostat Galvanostat PGSTAT302N (Metrohm, Netherlands) using a three-electrode setup consisting of Ag/AgCl as the reference electrode, platinum rod as the counter electrode, and the thin film samples as the working electrode in aCSF solution. To be able to extract the charge densities (μC cm^–2^) of the biointerfaces, 1 cm^2^ area of thin film samples was immersed in aCSF to obtain the current density (μA cm^–2^) for all the photocurrent measurements. aCSF solution was prepared by mixing the following materials in distilled water: 10 mM of 4-(2-hydroxyethyl)-1-piperazineethanesulfonic acid (HEPES), 10 mM of glucose, 2 mM CaCl_2_, 140 mM of NaCl, 1 mM of MgCl_2_, and 3 mM of KCl. After mixing, the pH of aCSF solution was adjusted to 7.4 by adding a stoichiometric amount of NaOH. Light pulses were applied *via* Thorlabs M450LP1 LED with 445 nm nominal wavelength, and the LED spectrum is provided in our previous study (Melikov et al., 2020b). The blue LED was driven with Thorlabs DC2200 - High-Power 1-Channel LED Driver. Newport 843-R power meter was used to measure the optical power of incident light on the devices. The illumination intensities were selected at the levels that can induce sufficient charge generation for stimulation of neurons (orders of μC cm^–2^) (Cogan, 2008).

### Electrochemical Analysis

Autolab Potentiostat Galvanostat PGSTAT302N (Metrohm, Netherlands) was used for electrochemical characterizations. For capacitance–voltage measurements, a three-electrode setup consisting of Ag/AgCl as the reference electrode, platinum rod as the counter electrode, and the thin film samples as the working electrode was used. The CV scans were monitored between certain voltage intervals for different device structures. During the measurement, the AC amplitude was kept 10 mV (RMS) to maintain the linearity of the response and measuring frequency was fixed at 1 kHz. The electrochemical impedance spectroscopy (EIS) was performed in frequency response analysis (FRA) potential scan mode. The blue illumination was applied to the devices while varying the frequency between 1 Hz and 10 kHz at 10 mV (RMS) AC voltage perturbation. The fitting of the responses was performed in NOVA software to extract the electrical parameters.

### Primary Neuron Isolation

All experimental procedures have been approved by the Institutional Animal Care and Use Committees of Koç University (Approval No: 2019.HADYEK.023) according to Directive 2010/63/EU of the European Parliament and of the Council on the Protection of Animals Used for Scientific Purposes. Hippocampal regions were extracted from decapitated E15-E17 Wistar Albino rats and were placed immediately in ice-cold Hank’s Balanced Salt Solution (HBSS, Thermo Fisher Scientific, MA, United States). The hippocampi were incubated in 0.25% Trypsin-EDTA solution (Thermo Fisher Scientific, MA, United States) with 2% DNase-I supplement (NeoFroxx, Einhausen, Germany) for 20 min in a 37°C incubator. Then, the cells were centrifuged, and the supernatant was changed with Dulbecco’s Modified Eagle Medium/Nutrient Mixture F-12 (DMEM/F12 Thermo Fisher Scientific, MA, United States) supplemented with 10% fetal bovine serum (FBS, Heat Inactivated, GE Healthcare, IL, United States) and 1% penicillin/streptomycin (Thermo Fisher Scientific, MA, United States). DMEM/F12 was removed, and Neurobasal Medium (NBM, Thermo Fisher Scientific, MA, United States) supplemented with B27, L-glutamine, β-mercaptoethanol, and glutamate (Thermo Fisher Scientific, MA, United States) was added to the cell pellet. The cells were triturated and were passed through a 70 μm cell strainer. The homogenous cell solution was seeded in poly-D-lysine (PDL, Sigma-Aldrich, MO, United States) coated substrates. After 3-day incubation of cells on substrates in a 37°C incubator with 5% carbon dioxide, the media of the cells on substrates were changed with NBM supplemented with cytosine arabinoside (Sigma-Aldrich, MO, United States) to inhibit growth of glial cells. After 24-h incubation with cytosine arabinoside, the media were changed with NBM and the substrates with the hippocampal neurons were used for experiments.

### Biocompatibility Assay

MTT viability assay was applied to investigate cell viability of primary hippocampal neurons on the biointerfaces. The neural growth medium was prepared by using B27 supplemented Neurobasal medium. MTT cell viability assay (ab211091, Abcam, Cambridge, UK) was utilized to evaluate biocompatibility of our biointerface. The devices were sterilized first by cleaning with 70% ethanol followed by air-drying. The surface was further sterilized under UV irradiation for 30 min. Substrates were placed in wells of the six-well plates. Primary hippocampal neurons were seeded (5 × 10^5^ cells per sample) on the substrates in B27 supplemented Neurobasal medium as described above and incubated in the neuron growth medium for 48 h after cytosine arabinoside supplemented neurobasal medium removal. After 48 h incubations, the media were replaced with 1 ml of MTT solution (5 mg/ml in PBS, pH = 7.4) and 4 ml of NBM mixture per well. Then, for an additional 4 h, the cells were incubated at 37°C and 5% CO_2_ atmosphere. The medium was vacuumed from each well and substrates were transferred to an empty six-well plates. In each well, 1:1 mixture of DMSO and ethanol was added to dissolve the formazan crystals. The solution was transferred to a 96-well plate, and the absorbance was measured at 570 nm light with Synergy H1 Micro-plate Reader (Bio-Tek Instruments). The relative cell viability was calculated as follows: viability = (OD_*sample*_/OD_*control*_) × 100. The optical density (OD) of the sample was obtained from the cells grown on a photoelectrode, and the OD of control was obtained from the cells grown on the ITO substrates.

### Immunofluorescence Staining and Imaging

Primary hippocampal neurons (5 × 10^5^ cells per sample) were seeded as explained above on ITO control substrate and the biointerface. The samples with neurons were fixed by 4% paraformaldehyde immediately after primary hippocampal neuron isolation protocol or incubated for 14 days with regular medium changes at 37°C in cell culture incubator. After 14-day incubation, the primary hippocampal neurons were also fixed by 4% paraformaldehyde and washed three times with PBS-T (Phosphate Buffered Saline, 0.1% Triton X-100). Cells were blocked in PBS solution containing 5% BSA (Bovine Serum Albumin) and 0.1% Triton X-100. Samples with primary hippocampal neurons were incubated with rabbit anti-NeuN antibody (ab177487, Abcam, Cambridge, United Kingdom) overnight, for neuron characterization, and washed three times with PBS-T. Then, samples with primary hippocampal neurons were incubated with goat anti-rabbit IgG H&L Alexa Fluor 555 (4413, Cell Signaling Technology, MA, United States) for fluorophore marking of anti-NeuN primary antibody for 90 min at 37°C. For visualization of the cytoskeleton, primary neuron samples were also incubated with FITC-conjugated phalloidin antibody (P5282, Sigma Aldrich, MO, United States) for 90 min at 37°C. All samples were washed three times with PBS-T and then mounted with DAPI supplemented mounting medium (ab104139, Abcam, Cambridge, United Kingdom) to observe nuclei. Finally, immunofluorescence imaging was done using a florescence light microscope (DMi8 S, Leica, Wetzlar, Germany).

### Electrophysiology Recordings

Single-cell electrophysiology experiments were performed using EPC 800 Heka Elektronik patch-clamp amplifier in whole-cell configuration. The preparation of aCSF is provided in section “Photoresponse Analysis”, and biointerfaces were electrically floating in aCSF, meaning that no wire is connected to the photoelectrodes. Photovoltaic QD/ZnO and TiO_2_/QD architectures for type I and type II biointerfaces, respectively, act as current-generating active electrodes. ITO back contact serves as the return electrode. Throughout the manuscript, “neural membrane” refers to the “free membrane” as defined in a previous study (Schoen and Fromherz, 2007). Transmembrane voltage is defined as the electrical potential difference between the intracellularly recorded voltage at the patched membrane region with respect to a distant reference electrode placed in the extracellular medium. Transmembrane voltage measurements were taken in current clamp mode while applying light pulses differing between 5 and 200 ms *via* an LED source (nominal wavelength: 445 nm; optical power density: 2 mW mm**^–^**^2^ corresponding to the minimum value that can evoke repetitive action potentials). The patch pipette resistance of 8–10 MΩ was used for the experiments. The pipettes were filled with an intracellular medium, which consists of 140 mM KCl, 2 mM MgCl_2_, 10 mM HEPES, 10 mM ethylene glycol-bis(β-aminoethyl ether)-N,N,N’,N’-tetraacetic acid (EGTA), and 2 mM Mg-ATP dissolved in distilled water. The pH of the intracellular solution was adjusted to 7.2–7.3 by adding a stoichiometric amount of KOH. Patch pipette and cells were monitored through a digital camera integrated with the Olympus T2 upright microscope.

## Results

### Quantum Dot Properties and Biointerface Design

The search for toxic-heavy-metal-free QDs has led to the synthesis of QDs made of III-V semiconductors [such as InP and AlSb (Bahmani Jalali et al., 2019b)]. Compared to II-VI QDs, which have large Phillips ionicity, III-V QDs are more robust in terms of optical stability due to the high covalency (lower Phillips ionicity) of their structure (Bharali et al., 2005; Chen et al., 2020). InP is one of the most widely studied III-V QD, and it has no intrinsic toxicity (Xie et al., 2007; Yong et al., 2009; Tamang et al., 2016; Wegner et al., 2019). Based on these reasons, we decided to use InP core and InP/ZnS core/shell QDs as the photoactive layer of our biointerfaces.

**FIGURE 1:**
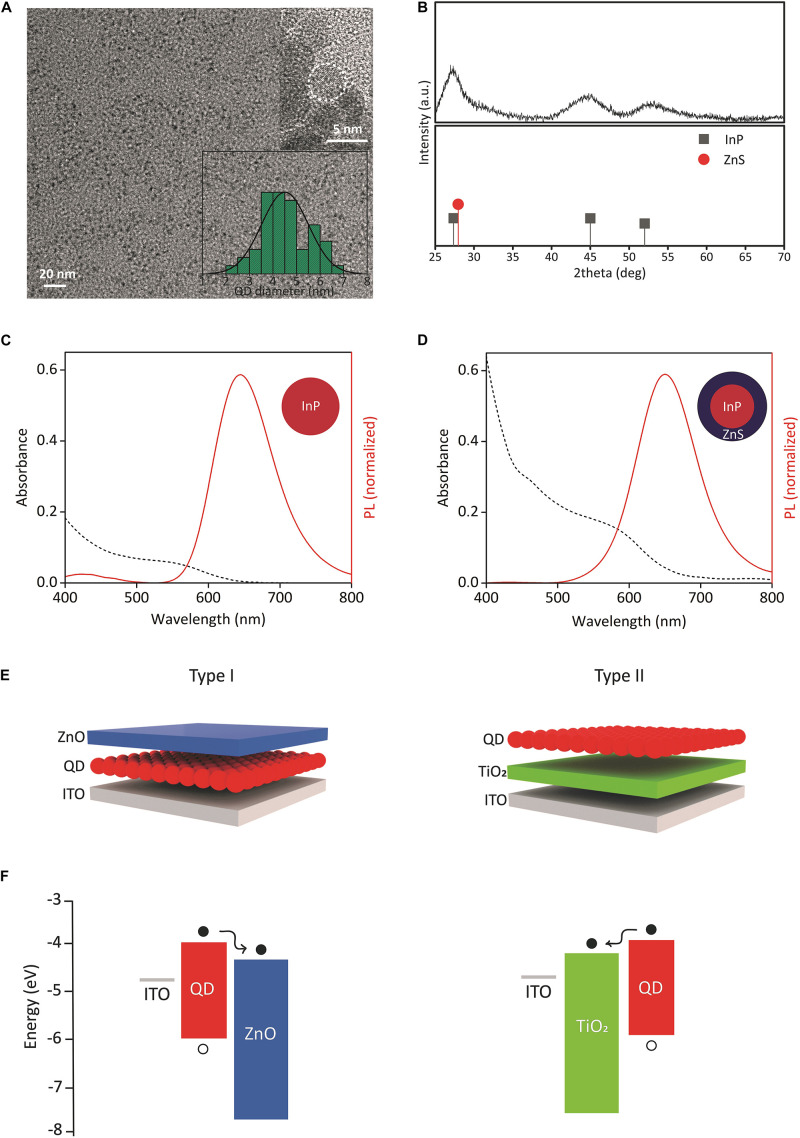
Structural and optical properties of QDs, device structures, and energy diagrams. **(A)** TEM image of InP/ZnS core/shell QDs. Inset shows the corresponding size distribution and HRTEM image. **(B)** XRD pattern of InP/ZnS core/shell QDs. The XRD peak positions of zinc blende bulk InP (JCPDS No. 32-0452) and bulk ZnS (JCPDS No. 80-0020) were shown. **(C,D)** Absorption and emission spectrum of InP core and InP/ZnS core/shell QDs, respectively. **(E)** (Left) type I and (right) type II biointerface configurations. Either InP core or InP/ZnS core/shell QDs were used as QD layer. **(F)** Energy band diagrams of (left) type I and (right) type II biointerfaces. The energy levels were taken from our previous study and literature (Pattantyus-Abraham et al., 2010; Yang et al., 2012; Karatum et al., 2019). The displacement of electrons (filled circles) and holes (empty circles) was shown.

We synthesized InP core QDs *via* hot injection method and grew ZnS shell for the formation of InP/ZnS core/shell nanostructure (Bahmani Jalali et al., 2018a). The transmission electron microscopy (TEM) analysis of InP core and InP/ZnS core/shell QDs (Figure 1A and Supplementary Figures 1, 2) shows an increase in the mean particle size from 3.2 nm to 4.5 nm diameter, indicating the formation of ZnS shell with a thickness of 0.65 nm and leading to a red shift in the PL spectrum. Moreover, the powder X-ray diffraction (XRD) analysis shows the crystal structure of the QDs by indicating zinc-blende crystal structures for InP and ZnS, respectively (Figure 1B).

We next investigated the optical properties of QDs. Figures 1C,D show the absorption and PL spectrum of the synthesized InP QDs and InP/ZnS QDs at the same concentration level (60 mg/ml), respectively. Both QDs absorb visible spectrum up to the red spectral region, and InP/ZnS QDs have higher absorbance than InP. In an integrated sphere system, the PL quantum yields (PL QY) of InP and InP/ZnS QDs were measured as 3 and 18%, respectively. Six-fold increase in quantum yield indicates the successful passivation of non-radiative recombination sites such as surface trap states (Chibli et al., 2011).

Using the synthesized QDs, we fabricated biointerfaces by solution-processing the constituent layers. The biointerfaces were fabricated in two different configurations, called type I and type II (Figure 1E). The device structures of type I and type II biointerfaces, and their corresponding energy band diagrams are presented in Figures 1E,F, respectively. ZnO and TiO_2_ nanoparticles in the device structures serve the purpose of blocking holes and controlling the electron movement, i.e., the photocurrent direction within the devices. Since the high annealing temperature of TiO_2_ might damage QD layer, ZnO was used as the top layer in type I device structure.

### Photoelectrical Performance of Biointerfaces

**FIGURE 2:**
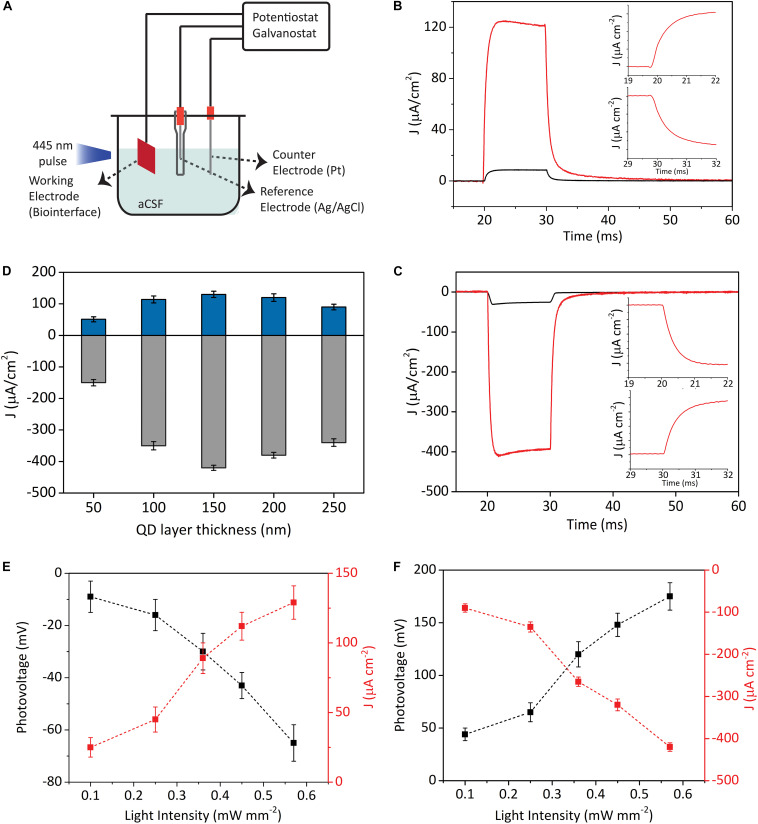
Characterization of the photoelectrical performance of biointerfaces. **(A)** Three-electrode electrochemical photocurrent measurement setup. **(B,C)** Photocurrent density of type I **(B)** and type II **(C)** devices in biological medium (illumination: blue LED at the wavelength of 445 nm, 10 ms pulse width, 0.57 mW mm**^–^**^2^ optical power density). Black lines represent when InP core QDs are used as the photoactive layer, while red lines show when InP/ZnS core/shell QDs are used as the photoactive layer. Inset **(B,C)** top: Zoomed onsets of photocurrents. Inset **(B,C)** bottom: Zoomed offsets of photocurrent. **(D)** Photocurrent densities of type I (blue bars) and type II (gray bars) biointerfaces as a function of photoactive layer thickness (Mean ± SD for *N* = 8). **(E,F)** Photovoltage and photocurrent density responses of type I **(E)** and type II **(F)** devices under different light intensities (illumination: blue LED at 445 nm, 10 ms pulse width) (Mean ± SD for *N* = 8).

Charging/discharging dynamics and the maximum photovoltage produced by the biointerfaces are important parameters to understand their light-triggered neuromodulation potential. Figure 2A shows the electrochemical setup for the characterization of the InP QD-based biointerfaces. We place the electrodes in aCSF, which is commonly used as an extracellular solution for neural tissues and electrophysiology (see section “Photoresponse Analysis” for the preparation of aCSF) (Lacour et al., 2010; Williamson et al., 2015). We measure their photocurrent and photovoltage *via* a three-electrode setup under light illumination with LED light source (445 nm nominal wavelength, optical power density ranging between 0.1 mW mm**^–^**^2^ and 0.57 mW mm**^–^**^2^) (Figure 2A). As it is evident from the electron migration directions shown in Figure 1F, we observe oppositely directed photocurrents in type I and type II devices. This is because the ZnO layer blocks the photogenerated holes at the QD layer from moving to the surface, which results in electron accumulation on the ZnO–electrolyte interface. In contrast, photogenerated holes are blocked by the TiO_2_ layer in type II devices, which causes hole accumulation on the QD–electrolyte interface. Thus, by properly engineering the band alignment of the constituent materials, we can control the direction of electron flow and the type of charge that will accumulate on the device–electrolyte interface. In that sense, type I and type II biointerfaces will generate opposite polarity photocurrents and reverse membrane potential variation; in other words, type I biointerface will bring membrane potential to more negative values (hyperpolarization) and type II biointerface will increase membrane potential (depolarization).

Figures 2B,C demonstrate the photocurrent density of the two types of devices incorporated with either InP core QDs or InP/ZnS core/shell QDs as the photoactive layer. In type I and II devices, we observe 10-fold and 13-fold higher photocurrent levels for the core/shell QDs compared to biointerfaces with core QDs, respectively. We ascribe this to the following two reasons: (i) InP/ZnS core/shell QDs have higher absorbance compared to InP QDs (Figures 1C,D), which leads to higher number of photogenerated excitons in the InP/ZnS layer compared to InP layer; (ii) decreasing the number of trap states by successful shell passivation of the InP core, which is supported by quantum yield enhancement, leads to higher currents. Due to stronger photocurrent generation, we decided to use InP/ZnS core/shell QDs inside type I and type II devices.

Different than capacitive double layer charging mechanism, the charging/discharging dynamics of photoelectrochemical current generation mechanism is dependent on the rates of electron transfer at electrode–electrolyte interface and the arrival rate of reaction ions to the interface (Merrill et al., 2005). Capacitive biointerfaces have fast charging dynamics with rise times on the order of tens or hundreds of microseconds (Ciocca et al., 2020; Han et al., 2020), whereas the decay times might be in milliseconds range (Jakešová et al., 2019). On the other hand, faradaic devices have typically longer rise/fall times due to the slower charging–discharging kinetics governed by electron transfer rate and availability of ions at the reaction site (Merrill et al., 2005; Bahmani Jalali et al., 2018b, 2019a). In this context, the photocurrents in Figures 2B,C rise to their maximum levels and falls back to their steady-state levels in less than 3 ms (insets of Figures 2B,C), which presents suitable charging/discharging dynamics for typical neuromodulation frequencies varying from few Hz to tens of Hz (Cogan, 2008) (the photoresponses of the biointerfaces for 5 ms and 1 ms pulses can be seen in Supplementary Figure 3).

We next investigated the current densities of our biointerfaces under illumination with different optical power densities and the resulting photovoltages (Figures 2E,F). The type I and type II biointerfaces can produce more than 25 mV photovoltage under optical power density of 0.1 mW mm**^–^**^2^. For the intensity of 0.57 mW mm**^–^**^2^, type I and type II biointerfaces produce -65 ± 7 mV and 175 ± 13 mV (Mean ± SD, for *N* = 8) photovoltages under 10 ms pulse, respectively. These numbers are promising for potential photostimulation applications considering the reported photovoltage values in a previous QD-based study that can evoke neural activity (Bareket et al., 2014), and also previous organic semiconductor-based biointerface studies that reported similar or lower photovoltage values and still effectively stimulate neurons (Gautam et al., 2014; Ciocca et al., 2020; Leccardi et al., 2020).

Moreover, the integrated area under the photocurrent transients is an important metric in terms of showing the charge injection quantities of the biointerfaces. We calculated the peak charge injection levels of type I biointerfaces as 1.29 μC cm**^–^**^2^, and type II biointerfaces as 4.12μC cm^–2^, which are at similar levels with the threshold charge density values of neural prostheses (Cogan, 2008).

### Photocurrent Maximization via Device Engineering

The effect of photoactive layer thickness on optoelectronic device performance has been investigated in the literature, especially for solar cells (Johnston et al., 2008; Kramer and Sargent, 2014; Yang et al., 2016; Krishnan et al., 2019). Regarding that, however, it is possible that the optoelectronic devices working in the biological medium have different dynamics. Indeed, our biointerfaces operate in aCSF, which consists of certain physiological ions and agents such as K^+^, Na^+^, Cl^–^, HEPES, and glucose dissolved in deionized water. Operation in such medium will result in different values of charge carrier parameters (e.g., mobility and diffusion length) compared to the investigated cases in the literature. Thus, the examination of the dependence of the biointerface performance to photoactive layer thickness in the biological medium provides valuable insight for photoactive stimulation devices.

Figure 2D shows the current density responses of type I and type II devices in aCSF medium for different photoactive layer thicknesses. We observe the same behavior in both types of devices, in which the photocurrent first increases up to certain photoactive layer thickness. Further increasing the thickness causes photocurrent to decrease. In other words, there is an optimum thickness, which results in the maximum photocurrent generation from the devices. The optimum thickness strongly depends on the depletion width and the minority carrier diffusion length. Depletion width is the region that the photogenerated charges are efficiently extracted. The generated charge carriers within a diffusion length of the space charge layer are also harvested with a high probability. If the photoactive layer is thicker than the optimum thickness, extracted charges recombine in the neutral region, which decreases the extraction efficiency. On the other hand, thinner photoactive layer is disadvantageous due to insufficient absorption.

To investigate the internal operation of our devices, we conducted Mott–Schottky analysis and electrical impedance spectroscopy (EIS), which together allow us to investigate the charge-carrier dynamics and calculate the depletion width and minority carrier diffusion length (Melikov et al., 2020b). Mott–Schottky analysis can be applied to the devices that contain a semiconductor–semiconductor junction in which one semiconductor is much more doped than the other one (Chang et al., 2013). In such a device, the depletion layer capacitance can be measured as a function of bias. The measured capacitance **(C)** and applied bias **(V)** are correlated to each other with the following expression (Willis et al., 2012):

(1) $$C^{-2}=\frac{2\,\left(V_{b\,i}-V\right)}{A^{2}\,q\,\varepsilon \,\varepsilon_{0}\,N}$$

where *V*_*bi*_ is the built-in voltage, *A* is the device area, *q* is the elementary charge, ε is the dielectric constant of the material, ε_0_ is the permittivity of free space, and *N* (*N_a_* for acceptor type, *N_d_* for donor type) is the doping concentration of the material.

**FIGURE 3:**
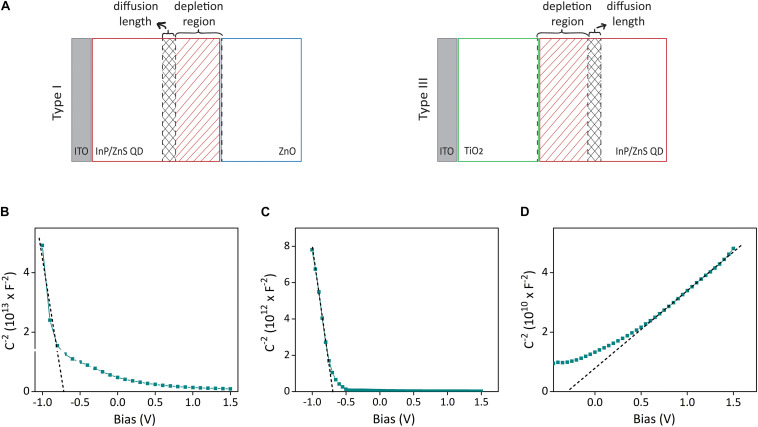
Depletion layer and diffusion width analysis of biointerfaces for photocurrent maximization. **(A)** The schematic showing the formation of depletion width and diffusion length on the photoactive layer, which both contribute to the photocurrent production. **(B–D)** Mott–Schottky capacitance–voltage analysis of ITO/QD, ITO/TiO_2_, and ITO/ZnO devices in aCSF medium with three-electrode configuration. Dashed lines show the linear fit.

Equation (1) and the individual capacitance–voltage measurements of ITO/QD, ITO/TiO_2_, and ITO/ZnO devices in aCSF solution (Figures 3B–D) yielded the carrier concentrations of QD, TiO_2_, and ZnO as *N*_*a*_ = 7.4 = 10^16^*cm*^−3^, *N*_*a*_ = 1.3 = 10^18^*cm*^−3^, and *N*_*d*_ = 6.5 = 10^20^*cm*^−3^, respectively. The fact that the doping concentrations of ZnO and TiO_2_ are much higher than the doping concentration of InP/ZnS QD indicates the formation of a space charge layer in the QD–ZnO and TiO_2_–QD junction. The presence of a depletion layer can also be inferred from the Mott–Schottky analysis of type I and type II devices, which both show bias dependent capacitance behavior (Supplementary Figure 4). It also implies that the depletion width will be predominantly in the QD layer in both types of devices. Thus, we can show the formation of depletion width and minority carrier diffusion length on the device schematic as in Figure 3A. Since ZnO and TiO_2_ have very low absorption in the blue spectral region due to their large band gaps, their contribution to the photocurrent production is negligible. Therefore, we can disregard the diffusion length in those layers. As the individual Mott–Schottky analysis of the QD layer revealed, the minority carrier in the InP/ZnS QD layer is electrons. As a result, we need to obtain the depletion width extending into the photoactive layer, and electron diffusion length for type I and type II devices. The depletion width (*w*) extending into the QD layer can be determined from the following relation (Kramer and Sargent, 2014):

(2) $$w_{1}={\left(\frac{2\,N_{2}\,\varepsilon_{1}\,\varepsilon_{2}\,\left(V_{b\,i}-V\right)}{q\,N_{1}\,\left(\varepsilon_{1}\,N_{1}\,\varepsilon_{2}\,N_{2}\right)}\right)}^{\frac{1}{2}}$$

whereε_1_ and ε_2_ are the permittivity of side 1 and side 2 (side 1 is taken as InP/ZnS, side 2 is ZnO in type I, TiO2 in type II devices), and *N_1_* and *N_2_* are the doping concentrations of side 1 and side 2. Extracting the built-in voltage of type I and type II devices from the capacitance–voltage plots in Supplementary Figure 4, equation (2) yields the depletion width of type I structure as 122 nm and type II structure as 94 nm.

To find the electron diffusion length, we conducted EIS analysis to type I and type II electrodes (Supplementary Figures 5A–D). By fitting the EIS plots with an equivalent circuit (Supplementary Figure 5E) and extracting the electrical parameters obtained from the fitted circuit, the electron diffusion length in type I and type II devices was determined as 43 nm and 91 nm, respectively (Table S1). Consequently, the sum of depletion width and diffusion length is 165 nm for type I and 185 nm for type II devices, which both match on the order of magnitude with the photoactive layer thickness that maximizes the photocurrent (150 nm) in Figure 2D.

### Stability and Biocompatibility of Biointerfaces

**FIGURE 4:**
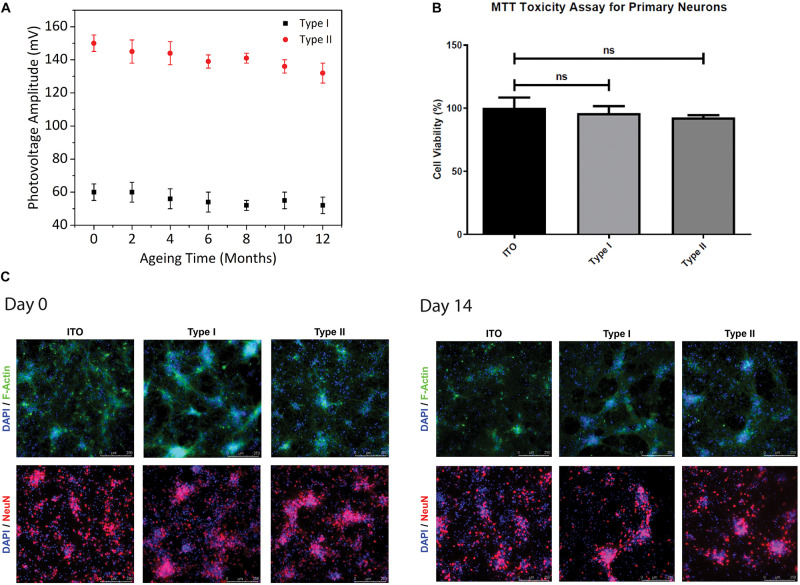
Stability and biocompatibility of biointerfaces. **(A)** Photovoltage measurements of type I and type II biointerfaces in an accelerated aging test for aging period of 12 months (Mean **±** SD, for *N* = 4). **(B)** Cell viabilities of primary hippocampal neurons cultured on type I and type II biointerfaces obtained from MTT biocompatibility assay analysis. Data was presented in a column graph plotting the mean with the standard deviation (Mean ± SD) (Four technical replicates were used in each of the three different experiments). Unpaired, two-tailed t-test was performed to determine the level of significance and * shows *p* < 0.05, which is considered as statistically significant and “ns” indicates a statistical non-significant difference. **(C)** Immunofluorescence images of primary hippocampal neurons and glia on type I, type II biointerfaces, and ITO controls at Day-0 and Day-14 after primary neuron isolation protocol. Primary hippocampal neurons co-stained with DAPI (blue), a DNA marker, Anti-NeuN antibody (red), a neural nucleus marker, and Anti-F-actin antibody (green), a cytoskeleton marker (Scale bar = 250 μm).

To test the reproducibility of the signals, we performed accelerated aging test as reported in previous studies (Ferlauto et al., 2018; Han et al., 2021). We placed the biointerfaces in physiological solution aCSF and kept them at 87°C for 12 days. We measured the photovoltages of the biointerfaces each 48 h *via* three-electrode electrochemical setup in galvanostatic mode. Assuming body temperature of 37°C, acceleration factor *f* at 87°C corresponds to 32 (*f* = 2^Δ*t/10*^, Δt = 87 − 37 = 50), hence yielding the simulated period of 384 days (12 months). Both type I and type II biointerfaces preserved their performance for the period of 12 months with less than 15% decrease in photovoltage (Figure 4A).

Although the biocompatibility of InP-based QDs was studied in detail (Yong et al., 2009; Lin et al., 2015; Chen et al., 2018; Li et al., 2020), the effect of the biointerfaces used in this study on the viability and metabolic activities of primary neurons should be quantified for their potential use as neurostimulators. We studied the biocompatibility of our biointerfaces by performing cell viability analysis *via* MTT toxicity assay and immunofluorescence imaging. The effect of the biointerfaces on metabolic activities of primary hippocampal neurons was assessed and compared with ITO control samples after 48-h incubation in the neuron growth medium (Figure 4B). The MTT results indicate that the biointerfaces did not have an adverse effect on cell viability of primary hippocampal neurons. Neurons grown on type I and type II biointerfaces demonstrate comparable levels of cell viabilities with respect to the reference ITO substrate, which is known as a biocompatible material for neural cells. No significant decrease on cell viability primary neurons is observed in type I and type II biointerfaces compared to ITO. Besides, immunofluorescence images of primary hippocampal neuron culture on type I, type II, and ITO control samples taken at day 0 and day 14 indicate the maintained cell viability and morphology (Figure 4C), which agrees with MTT assay results.

### Neural Photostimulation With Optimized Biointerfaces

**FIGURE 5:**
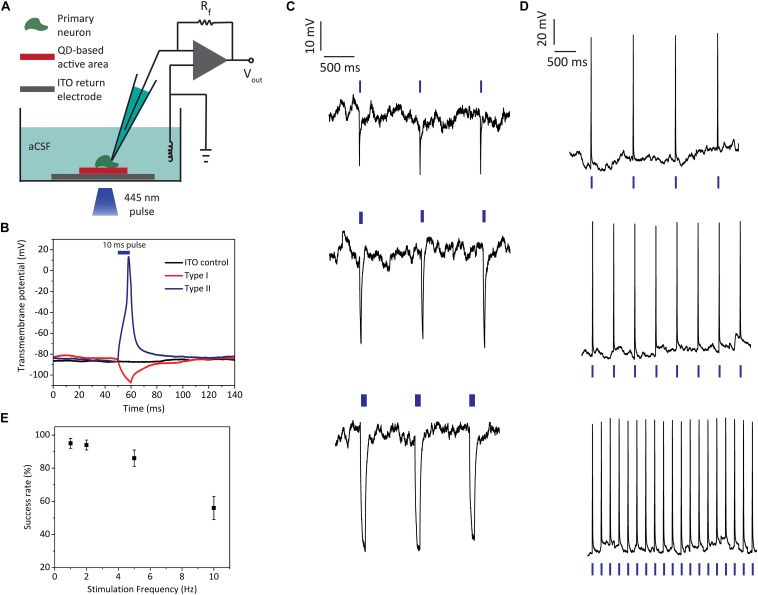
Light-induced electrophysiology recordings of primary hippocampal neurons cultured on biointerfaces. **(A)** Schematic of the single cell electrophysiology setup. **(B)** Transmembrane potential recordings of neurons on type I, type II, and ITO control samples (illumination: blue LED at 445 nm, 10 ms pulse width, 2 mW mm**^–^**^2^ optical power density; blue bar indicates the 10 ms “light on” interval). **(C)** Neural membrane recordings of hippocampal neurons on type I biointerfaces for 1 Hz stimulus with different pulse widths (top: 10 ms, middle: 50 ms, bottom: 200 ms) (illumination: blue LED at 455 nm, 2 mW mm**^–^**^2^ optical power density; blue bars indicate the “light on” intervals). **(D)** Neural membrane recordings of hippocampal neurons on type II biointerfaces for 1, 2, and 5 Hz stimulus (the membrane response to 10 Hz stimulus is shown in Supplementary Figure 7) (illumination: blue LED at 445 nm, 10 ms pulse width, 2 mW mm**^–^**^2^ optical power density; blue bars indicate the “light on” intervals). **(E)** Success rate of action potential firing for type II biointerfaces for 1, 2, 5, and 10 Hz stimulus frequencies (Mean ± SD, for *N* = 6).

We next conducted *in vitro* single-cell electrophysiology experiments with the type I and type II biointerfaces to show the light-induced effects on neural cell membranes under pulsed LED illumination (445 nm nominal wavelength, 2 mW mm**^–^**^2^ optical power density). The primary hippocampal neurons were cultured on top of our biointerfaces, and the transmembrane voltage changes on their cell membrane were measured *via* patch clamp setup in whole-cell configuration. Figure 5A shows the schematic of electrophysiology recording experiment with primary neurons. The QD/ZnO and TiO_2_/QD heterostructures for type I and type II biointerfaces serve as the active area that photogenerates charge carriers, while conductive ITO back contact serves as the return electrode in the stimulation experiments. Following the charge separation at the QD–ZnO or QD–TiO_2_ heterojunction, one type of charge carrier is moved to the electrode–electrolyte interface, giving rise to photoelectrochemical reactions with the electrolyte that leads to the photocurrent generation. The reactions occurring at the active area–electrolyte interface are balanced with the counter reactions taking place at ITO–electrolyte interface, completing the current loop (see Supplementary Material section “Characterization of Photoelectrochemical Processes” for details of electrochemical reactions taking place at the electrode-electrolyte interface).

In the single cell electrophysiology experiments, transmembrane voltage is defined as the electrical potential difference between the intracellularly recorded voltage at the patched membrane region and distant reference electrode placed in the extracellular medium. As expected from the reverse photocurrent directions of type I and type II biointerfaces, photoexcitation of the biointerfaces leads to countereffects on transmembrane voltages of primary neurons. Figure 5B shows the effect of type I and type II biointerfaces on neural transmembrane voltage together with ITO control sample when we illuminate them with 10 ms pulses. Type I biointerface hyperpolarizes the neural membrane, while type II biointerface depolarizes the membrane and evokes action potential. The neurons on ITO control sample did not show light-induced transmembrane potential change. We also checked repetitive photostimulation of neurons by applying consecutive pulses. Type I biointerfaces induce hyperpolarization of transmembrane voltage reproducibly *via* 1 Hz excitation (Figure 5C). In the same figure, we also observe the increase in the hyperpolarization amplitude as the pulse width is increased from 10 ms (5C top) to 50 ms (5C middle) and 200 ms (5C bottom). The hyperpolarization amplitude increased from 24 ± 3 mV for 10 ms to 34 ± 4 mV for 50 ms and 45 ± 6 mV for 200 ms (Mean ± SD, *N* = 6). This behavior is indicative of resistive coupling of the photocurrent to the neural membrane rather than capacitive coupling. One main advantage of resistive processes is their high charge capabilities (Merrill et al., 2005; Cogan, 2008), which makes them favorable for both direct electrical stimulation (e.g., iridium oxide electrodes) and optical stimulation interfaces (e.g., HgTe QD-based (Pappas et al., 2007) and silicon nanowire stimulators (Parameswaran et al., 2018)). This is reflected in the performance of type II biointerfaces, which can successfully elicit reproducible action potentials by depolarizing the neural membrane through continuous charge injection during the “light on” periods (Figure 5D). Photoexcitation of type II biointerfaces with 10 ms pulses (445 nm, 2 mW mm**^–^**^2^ optical power density) at 1, 2, and 5 Hz frequencies led to reproducible firing of primary neurons with success rates over 85%, while the spike rate is still over 50% for 10 Hz (Figures 5D,E) (see Supplementary Figure 7 for 10 Hz stimulus response).

## Discussion

**Quantum dots** have been one of the central nanomaterials for neural interfaces together with π-conjugated organic and silicon-based inorganic systems (Di Maria et al., 2018; Zimmerman and Tian, 2018). One of the major challenges of QD-based neural interfaces is the use of toxic heavy metal content (cadmium or mercury-based) QDs. InP-based QDs show a promising non-toxic alternative to be used for neural interfaces owing to the composition of III–V elements with covalent bonds in their structure and not containing highly toxic elemental compounds (Bharali et al., 2005). In addition to the previous reports showing the biocompatibility of InP-based QDs for both *in vitro* and *in vivo* (Yong et al., 2009; Lin et al., 2015; Bahmani Jalali et al., 2019a), our study showed the biocompatibility of InP QD-based type I and type II biointerfaces on primary hippocampal neurons *in vitro*, which are commonly used neural cell type to observe neurotoxicity. Moreover, the Bohr exciton radius of InP (∼9 nm) is larger than CdSe (∼5 nm), which gives a high-level controlling ability of electron and hole energy levels. Type I and type II heterostructures also offer another degree of freedom for wavefunction engineering for potential neuromodulation applications (Karatum et al., 2021).

The comparison of the photoelectrical performance of InP core and InP/ZnS core/shell photoactive layers has crucial importance as the shell deposition is an important practice for decreasing the cytotoxic effects of QDs, but little was known about the impact of shell coverage to the performance of QD based biointerfaces (Zimmerman and Tian, 2018). We demonstrated that shell growth facilitates substantial enhancement of photoelectrochemical current levels. In addition to the QD level control, their optoelectronic engineering offers the ability to demonstrate unconventional biointerfaces using non-radiative energy transfer, like in photosynthesis processes of plants (Bahmani Jalali et al., 2019a).

The photocurrent maximization procedure and agreement with the electrochemical measurements of the biointerfaces presented in this study show promise for future QD-based non-genetic neuromodulation studies. The electrophysiology experiments indicate the potential of the biointerfaces, demonstrating reproducible hyperpolarization and depolarization of primary neural membrane, which triggers neurons to fire light-induced action potentials. Besides, the light intensity levels used in the photostimulation experiments in this study are below the levels for the photothermal stimulation of neurons (Martino et al., 2015; Yoo et al., 2018).

## Conclusion

Our findings show that QD core/shell heterostructure, device configuration, choice of photoactive layer, and the thickness of the photoactive layer are all effective on the performance of photoelectric biointerfaces. The ability to control the direction and strength of the stimulation is possible through proper band alignment engineering, nanostructure engineering, and optimization of the photoactive layer thickness. Therefore, the systematic engineering of the device parameters and the QD nanostructure in this study leads to the fabrication of effective InP QD-based photoactive biointerfaces that can optically control the electrical activity of neurons.

## Data Availability Statement

The original contributions presented in the study are included in the article/Supplementary Material, further inquiries can be directed to the corresponding author.

## Ethics Statement

The experimental procedure on animals was reviewed and approved by Institutional Animal Care and Use Local Committee of Koç University (Approval No: 2019.HADYEK.023) according to Directive 2010/63/EU of the European Parliament and of the Council on the Protection of Animals Used for Scientific Purposes.

## Author Contributions

OK, MA, and SN designed the experiments. GE and HB conducted material synthesis and characterization. OK fabricated and characterized the devices. OK and RM performed photoresponse measurements and electrophysiology experiments. SBS performed electrochemical characterization. SS performed cell culture and biocompatibility experiments. EY performed primary hippocampal neuron isolation, biocompatibility assay, immunofluorescence staining, and imaging of primary hippocampal neurons. BU supervised the photoelectrochemistry experiments and interpreted the data. AS and IK supervised the cell culture and biocompatibility experiments and interpreted the data. ID prepared the optical setup. OK and SN wrote the manuscript with input from all other authors. All authors contributed to the article and approved the submitted version.

## Conflict of Interest

The authors declare that the research was conducted in the absence of any commercial or financial relationships that could be construed as a potential conflict of interest.
