# Supplementary material for: Nanoengineering InP Quantum Dot-Based Photoactive Biointerfaces for Optical Control of Neurons
Source: Front Neurosci. 2021 Jun 23;15:652608. doi: 10.3389/fnins.2021.652608 (PMC8260855; doi:10.3389/fnins.2021.652608)
Supplement: Supplementary file 1 [file Data_Sheet_1.docx]

Supplementary Material

# Transmission Electron Microscopy (TEM) images of the QDs


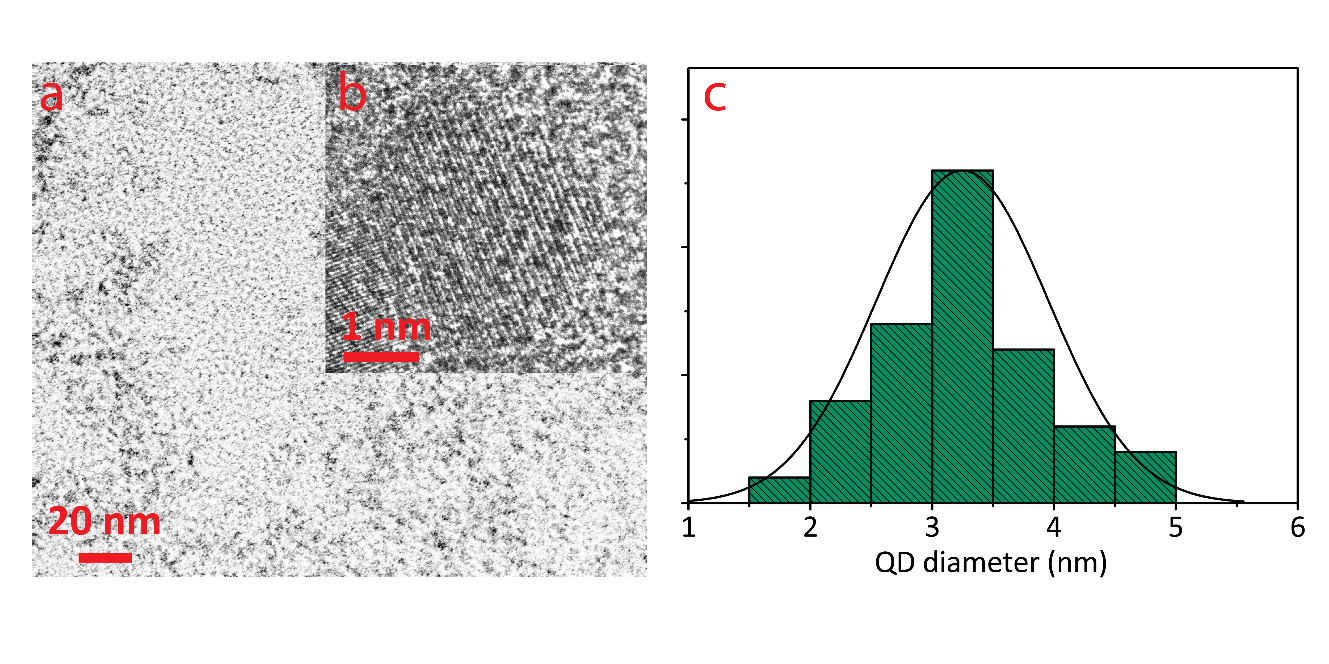


**Supplementary Figure 1.** a) Transmission Electron Microscopy (TEM) image of InP core QDs b) High resolution TEM (HR-TEM) image of InP QDs, and c) the corresponding size distribution (50 particles were counted).


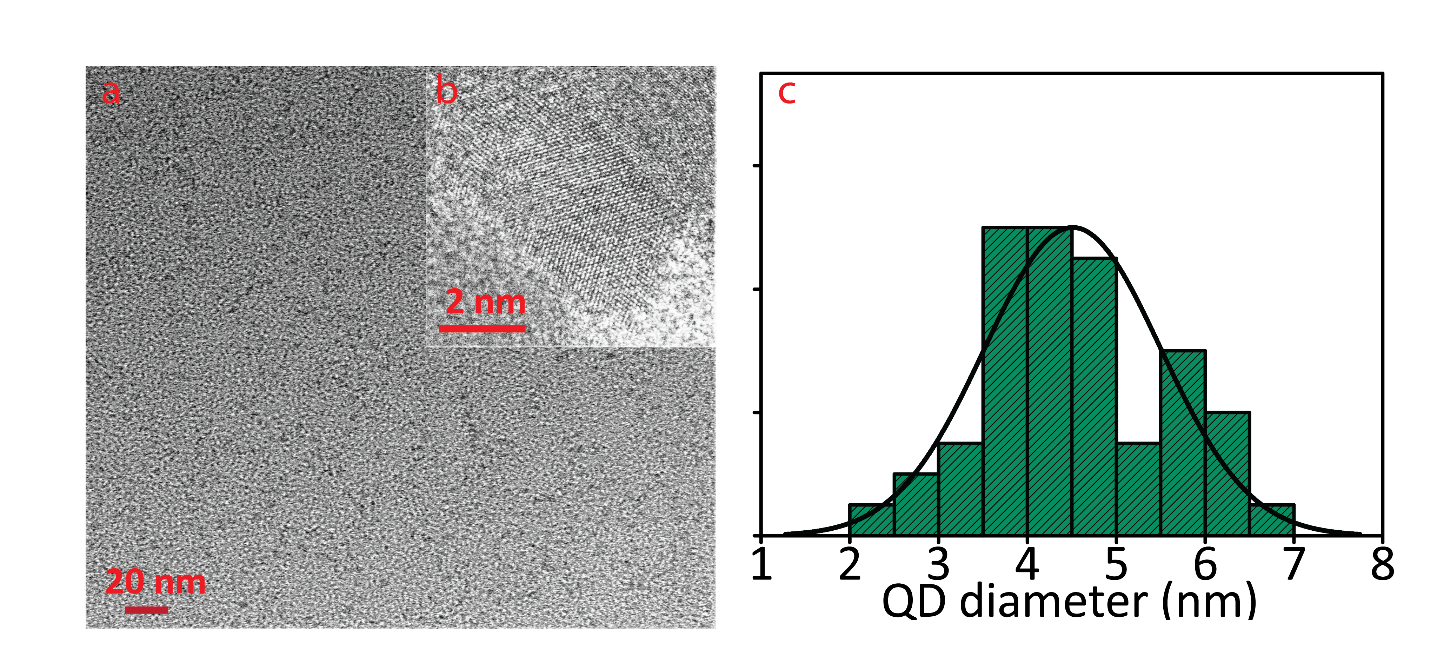


**Supplementary Figure 2.** a) Transmission Electron Microscopy (TEM) image of InP/ZnS core/shell QDs b) High resolution TEM (HR-TEM) image of InP/ZnS core/shell QDs, and c) the corresponding size distribution (50 particles were counted).

# Photocurrent Response of the Biointerfaces for Shorter Pulses


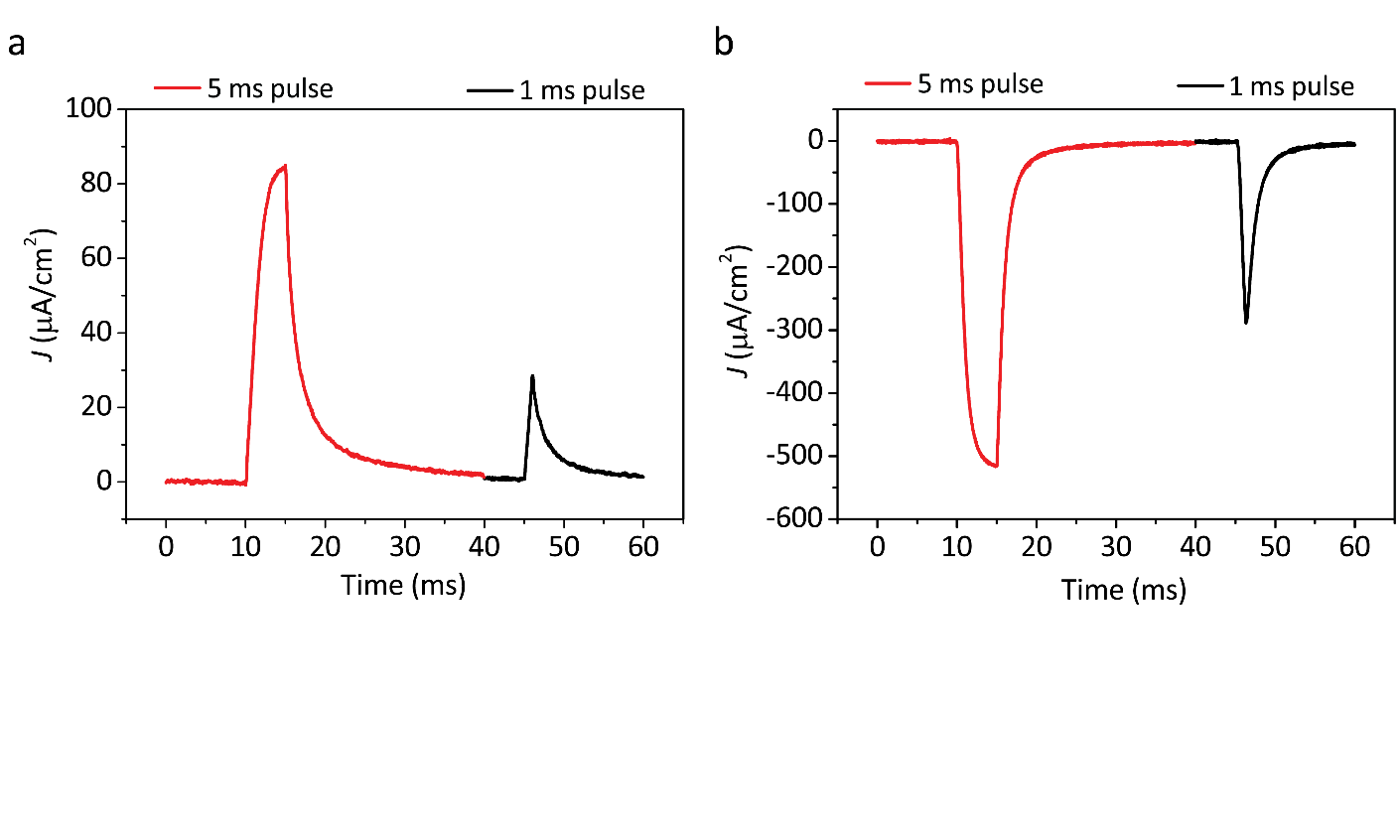


**Supplementary Figure 3.** The photocurrent responses of a) type I and b) type II biointerfaces for 5 ms and 1 ms light pulses (illumination: blue LED at the wavelength of 445 nm, 0.57 mW mm^-2^ optical power density).

# Electrochemical Characterization for Photocurrent Maximization


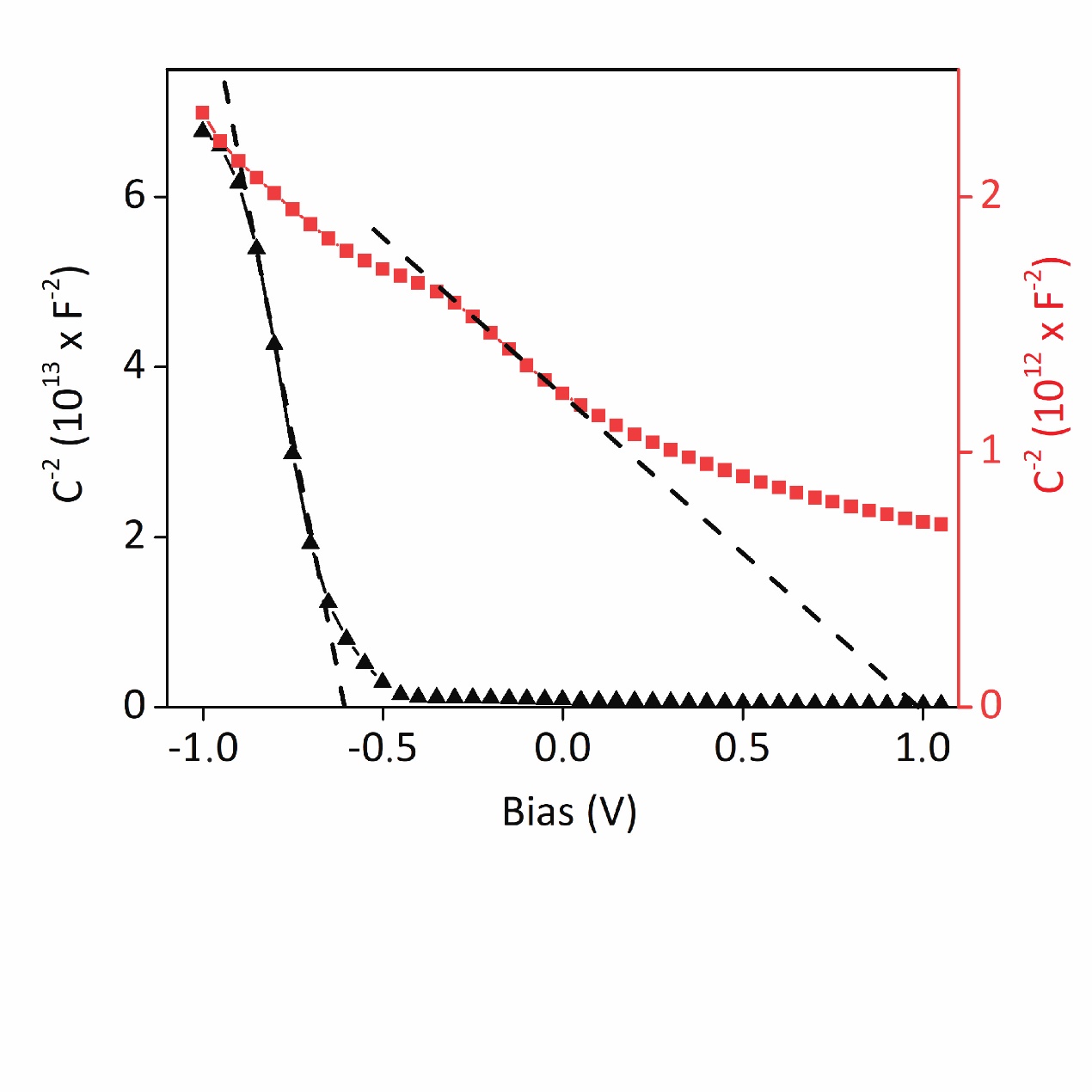


**Supplementary Figure 4.** Mott-Schottky analysis of whole devices. Capacitance-voltage plot of type I (red) and type III (black) devices in aCSF medium. The intersection point of the linear fits on the x-axis corresponds to the built-in voltage. Bias dependent capacitance behavior implies the presence of space charge layer at the semiconductor-semiconductor junctions for both types of devices.


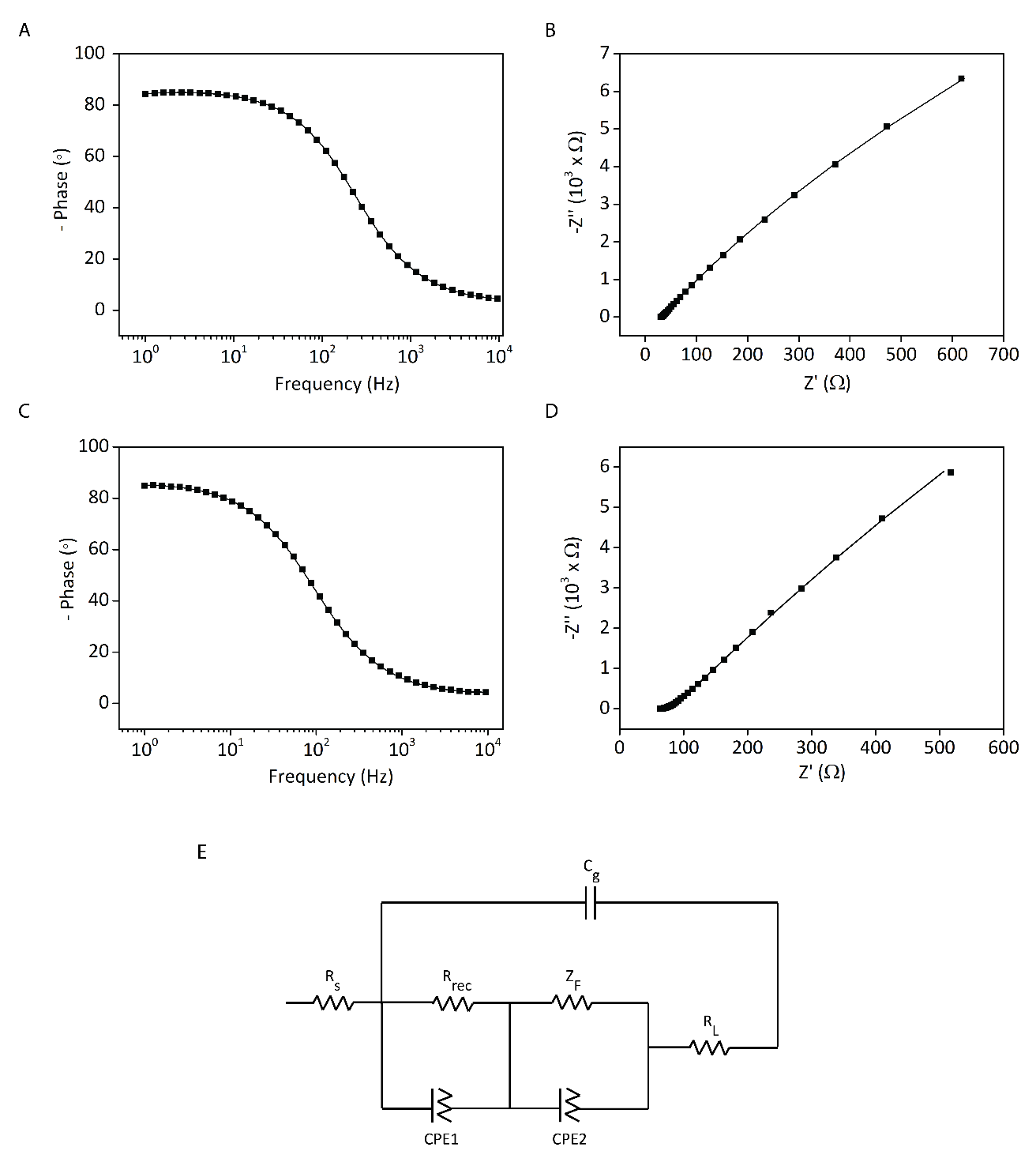


**Supplementary Figure 5.** Electrical impedance spectroscopy (EIS) measurements of type I and type III photoelectrodes. **(A)** Nyquist plot, **(B)** Bode phase plot of type I devices. **(C)** Nyquist plot, **(D)** Bode phase plot of type III devices. **(E)** Schematic of the fitted circuit diagram for both type I and type III devices. For A, B, C, D, measurement points are shown with the squares, and the corresponding fitting functions are shown with the lines.

Supplementary Table 1. Extracted parameters from electrochemical analysis. Resulting device parameters calculated using the EIS measurement outputs and the fitted circuit.

|  | Type I | Type II |
| --- | --- | --- |
| R_rec_ | $2.44 \Omega$ | $51.1 \Omega$ |
| Y_0_ | $178 \mu Mho$ | $4 \mathrm{mMho}$ |
| N | 0.847 | 0.367 |
| r_t_ | $27 \Omega$ | $54 \Omega$ |
| L | 122 nm | 92 nm |
| $C_{\mu}$ | $44 \mu F$ | $260 \mu F$ |
| $\tau_{n}$ | $0.107 \mathrm{ms}$ | $13.3 \mathrm{ms}$ |
| $\tau_{d}$ | $1.12 \mathrm{ms}$ | $14 \mathrm{ms}$ |
| D_n_ | $1.44 \times{10}^{-11}$ m^2^ s^-1^ | $4.6 \times{10}^{-13}$ m^2^ s^-1^ |
| $\mu_{n}$ | $9.22 \times{10}^{-29}$ m^2^ V^-1^ s^-1^ | $2.94 \times{10}^{-30}$ m^2^ V^-1^ s^-1^ |
| L_diff_ | 43 nm | 91 nm |

# Characterization of photoelectrochemical processes

To understand the faradaic reactions taking place in the electrode-electrolyte interface, we first measured the dark voltage (without illumination) of the biointerfaces in three electrode electrochemical setup via choronopotentiometry measurement. aCSF was used as electrolyte to mimic the stimulation conditions. The voltages of type I and type II biointerfaces with respect to Ag/AgCl reference electrode were measured as 2.2 V and 2.1 V, respectively. The fact that these voltages are higher than water electrolysis potential of 1.23 V implies that water electrolysis is a potential reaction that might be contributing to the photocurrent generation of the biointerfaces.

In order to identify whether any of the solutes of the aCSF is contributing to the photocurrent generation instead or in addition to water. We prepared six different aCSF solutions, each one is deficient of only one component. Then, in each solution, we measured the photocurrents of type I and type II biointerfaces (Supplementary Figure 6). The photocurrents for each biointerface drop significantly only in cases of NaCl removal or HEPES removal. Since NaCl is the predominant component that provides conductivity of aCSF, the photocurrent decrease in case of NaCl removal can be due to the decreased conductivity of electrolyte. To check that, we increased the KCl amount (to 140 mM) in the solution without NaCl and measured the photocurrent. Photocurrent increased to similar levels with full aCSF case, confirming that the reason for photocurrent drop in case of NaCl removal is due to the decreased conductivity. As a result, we can conclude that the faradaic photocurrent originates from the reactions involving water electrolysis and HEPES.

HEPES is known to be oxidizable near 1.5V vs. RHE (Lai et al., 2019). This represents a somewhat higher yet comparable overall potential when coupled to water reduction. Therefore, the overall oxidation current is split between HEPES and water and the coupled reduction current is predominantly water reduction.





**Supplementary Figure 6.** Photocurrent measurements in six different solutions, each prepared by removing only one ingredient from aCSF. Photocurrent values (mean ± s.d. for N = 3) were normalized by taking full aCSF photocurrent as 100%. The values in circle represents the case where NaCl is removed but KCl concentration is increased to 140 mM.

# Current clamp recordings for 10 Hz photostimulation


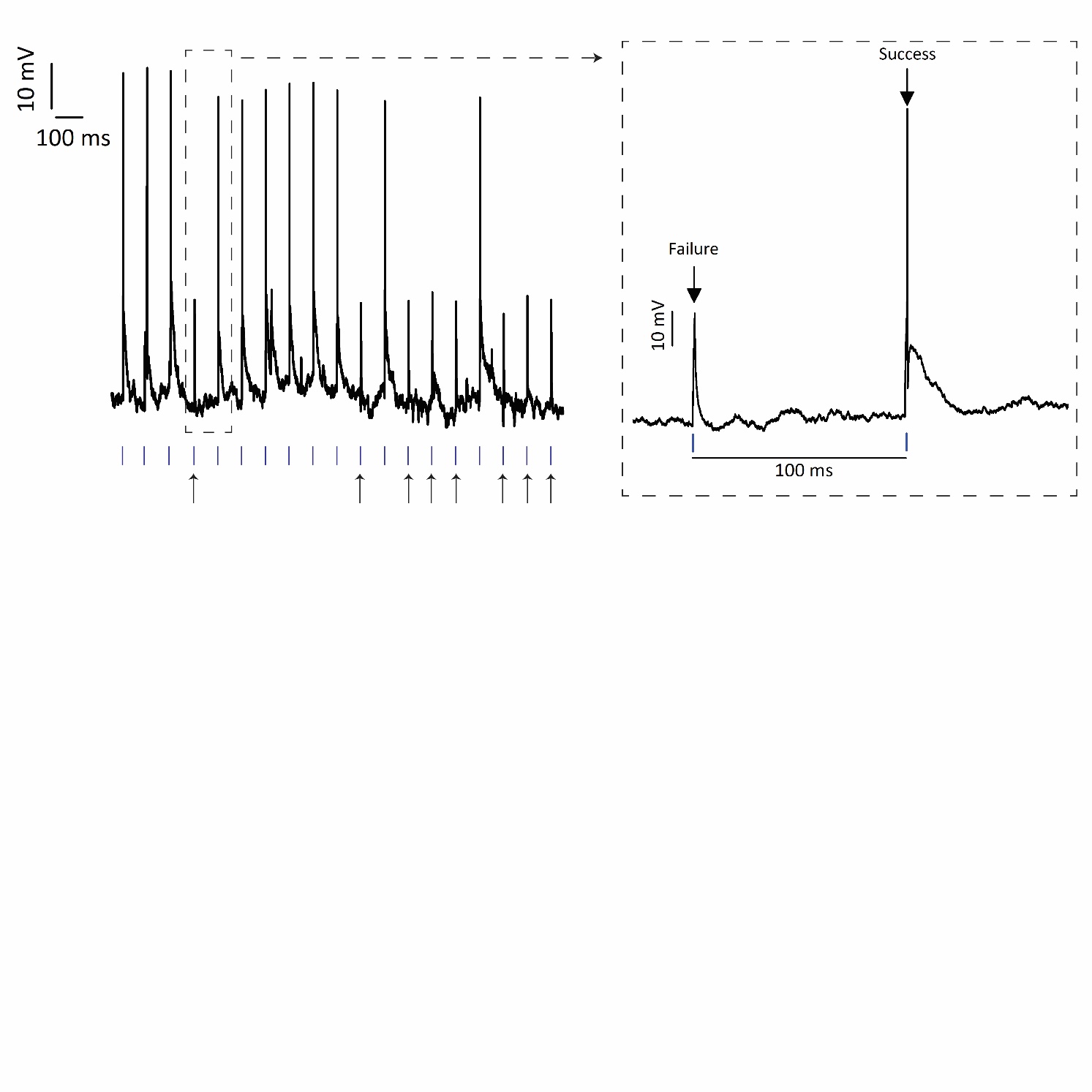


**Supplementary Figure 7.** 10 Hz stimulation of neurons on type II biointerfaces with 10 ms pulse (445 nm, 2 mW mm^-2^). Blue bars indicate the start of the light pulses. Arrows indicate the failed responses. Zoomed image of the dashed rectangle shows a failed stimulation pulse and a successful pulse that evoked an action potential.

# Intensity dependent current clamp recordings





**Supplementary Figure 8.** Current-clamp recording of hippocampal neurons in whole-cell configuration under different light intensities. Light is turned on at t = 50 ms (illumination: blue LED at 445 nm, 10 ms pulse width).

**References**

Lai, B.-C., Wu, J.-G., and Luo, S.-C. (2019). Revisiting background signals and the electrochemical windows of Au, Pt, and GC electrodes in biological buffers. *ACS Applied Energy Materials* 2**,** 6808-6816.
